# The impulsive online shopper: effects of COVID-19 burnout, uncertainty, self-control, and online shopping trust

**DOI:** 10.1186/s43093-022-00174-0

**Published:** 2022-12-08

**Authors:** Shunying Zhao, Qiang Yang, Hohjin Im, Baojuan Ye, Yadi Zeng, Zhinan Chen, Lu Liu, Dawu Huang

**Affiliations:** 1 School of Psychology, Jiangxi Normal University, 99 Ziyang Avenue, Nanchang, Jiangxi 330022 China; 2 School of Education Science, Jiaying University, Mei-Songong Road, Meizhou, Guangdong 514021 China; 3 School of Education, Jiangxi Normal University, 99 Ziyang Avenue, Nanchang, Jiangxi 330022 China; 4 Department of Psychological Science, University of California Irvine, 4201 Social and Behavioral Sciences Gateway, Irvine, CA 92697 USA; 5 School of Physical Education, Jiangxi Normal University, 99 Ziyang Avenue, Nanchang, Jiangxi 330022 China

**Keywords:** Impulse buying, COVID-19 burnout, Self-control, Uncertainty, Ambiguity, Trust

## Abstract

Consumerism during the COVID-19 pandemic has been characterized by impulsive buying. Using the theoretical lens of uncertainty avoidance and ego-depletion to identify the mediating mechanisms and moderating factors for online impulse buying, we surveyed young consumers across two relevant periods for high consumerism—the week preceding the 2021 Chinese Spring Festival (Study 1; February 4–9, 2021, *n* = 1495) and the weeks during and after the festival (Study 2; February 12 to March 2, 2021, *n* = 923). Perception of COVID-19 variant uncertainty was both directly and indirectly (via online shopping trust) positively associated with online impulse buying. COVID-19 burnout was consistently indirectly associated with online impulse buying via self-regulation and self-appraised impulsivity but inconsistently directly associated. Self-regulation was surprisingly positively associated with online impulse buying, possibly reflecting evidence of already depleted resources from prolonged regulatory exertion among high self-regulators. Self-appraised impulsivity negatively interacted with perception of COVID-19 variant uncertainty, suggesting that as trait impulsivity increases, individuals are less incentivized by peripheral drivers of online impulse buying.

## Introduction

Although the world has made strides in combatting COVID-19, the recent flurry of variants (e.g., *delta* [B.1.617.2], *omicron* [B.1.1.529]) has posed several uncertainties for the future. For instance, a resurgence of viral spread in the Spring of 2022 in China has resulted in Shanghai being placed under strict lockdown. In response to the pandemic and the administrative interventions that followed [23, 41], many consumers changed their behaviors, such as utilizing more online shopping mediums [46, 76] or compulsively buying products in panic [66, 84]. However, despite the attention given to compulsive buying [66], whether classic theories of the antecedents of impulse buying are translatable to online mediums amid the pandemic remains relatively understudied. Indeed, as the COVID-19 pandemic shuttered businesses and increased consumers’ exposure to e-commerce channels, investigation into online impulse buying is a critical topic of inquiry for both scholars and practitioners alike.

To investigate conceptual correlates of online impulse buying, we rely on the extant body of literature on uncertainty [25, 31, 45] and self-control [4, 13, 34, 73] to propose a moderated multi-mediation model. We posit that the perception of uncertainty of COVID-19 variants has a direct effect on online impulse buying and an indirect effect via online shopping trust. To capture the psychological consequences of a prolonged pandemic, we further examined the direct effect of COVID-19 burnout on online impulse buying as well as indirect effects via self-control (i.e., self-regulation and impulsivity). We further explored the possible moderating effects of self-control based on its common conceptualization as an individual trait.

### Impulsive buying

Impulsive buying is characterized as the unplanned and uncontrollable urge to buy goods [4, 74] that is motivated by both cognitive and affective factors [30], such as fear [12, 62] and disregard for consequences [74]. Failure of rational decision making and intrusion of irrational feelings and thoughts that more consumption will remedy negative states principally under impulsive buying [28, 78]. Harnish and Bridges [28] argue that irrational beliefs about avoidant coping and self-demoralization create a cycle of impulsive buying as a maladaptive outlet [50, 51]. The hedonic gratification that follows an impulsive purchase motivates the vast majority of consumers to engage in this behavior at least occasionally [30] which often results in guilt and diminished self-esteem afterward [28]. Within the context of COVID-19, the pandemic induces both affective and cognitive reactivity among individuals [95, 96], fueling changes in consumption behavior [15, 61].

During the infancy of the pandemic, several businesses were shuttered through government-mandated lockdowns and many consumers were newly introduced to online modalities of consumption [46, 61, 76]. Consequently, online impulse buying paralleled the rising use of e-commerce [15, 76]. Such trends were not surprising, however, as impulse buying has routinely been implicated to be a mode of coping with the sudden loss of control over one’s environment following disasters [22, 35, 36]. Thus, the current study investigates the conceptual drivers of online impulsive buying as a form of irrational coping using classic theories of uncertainty avoidance and self-control during a relevant social ecology of heightened distress.

### Perceived uncertainty of COVID-19 variants and online impulsive buying

Perceived uncertainty is the subjective appraisal of a situation’s ambiguous nature that inhibits one’s abilities to adequately assess probable outcomes [9]. Classical theories of uncertainty (e.g., *Uncertainty Reduction Theory*, *Motivation to Reduce Uncertainty Theory*) posit that individuals are motivated to utilize various strategies (e.g., seeking information) to increase clarity [7, 42, 56]. For simplicity, we refer to the broader body of these theories as the *uncertainty avoidance and reduction* (UAR) theory. UAR theory argues that novel stimuli without predictable outcomes breed discomfort due to the loss of one’s agentic control of their environment [25, 31, 45] and accordingly increase preference for behaviors with more predictable outcomes [33]. The emergence of novel health crises historically begets public angst, such as in the case of the 2009 H1N1 outbreak [82, 83] and most recently the COVID-19 pandemic [2, 86]. Even with the dissemination of vaccines in late 2020, the evolving state of COVID-19 and its variants (e.g., delta, omicron) presents a myriad of public health uncertainties.

Within the context of consumerism, consumers may look toward altering their buying behavior as a coping mechanism to regain some degree of personal control [4, 12, 87], particularly by increasing the rate of online impulsive consumption [10, 12, 75, 87]. With the availability of online only retailers (e.g., Amazon.com, Ebay, Alibaba) and many traditional retailers offering online options (e.g., Walmart + , Target Shipt), consumers have an unprecedented number of alternative channels to shop from to fulfill their desires [67]. Thus, online impulse buying may be a readily accessible outlet for coping [10, 16, 94] while successfully avoiding primary threat sources (e.g., physical proximity to others) [35, 43, 49, 77] and enjoying the immediate gratification from hedonistic purchases [22]. Indeed, the high degree of ambiguity at the start of the pandemic has been implicated to be a prime culprit for the unprecedented spikes in spending by consumers [62, 66]. Perceptions of COVID-19 uncertainty, however, are subject to wane with growing familiarity. To address this, the current study captured perceptions of uncertainty pertaining to *COVID-19 variants*, a relevant topic of concern at the time of data collection (February 2021) for the current study’s sample of Chinese consumers as China tackled its first wave of variants. As was the case with past public health crises, we posit the following:

#### Hypothesis 1

Perceived uncertainty of COVID-19 variants is positively associated with online impulse buying.

### Uncertainty & online shopping trust

Trust is a fundamental component in social exchanges [8] and has accordingly acted as the catalyst in maintaining positive buyer–seller relationships [39, 64]. Several past studies have routinely shown that trust is one of the most significant elements in online consumerism [85], increasing one’s intentions to shop online [32, 71, 80] by lessening associated perceived risks and fostering consumer confidence [3, 18, 29]. China’s Zero-COVID approach includes sudden government-mandated city lockdowns that shutter businesses and limit outside activities in response to even a few cases of COVID-19 [59]. Compared to physical retailers and stores, online retailers may therefore be a more consistent and trustworthy outlet for consumerism amid continued possibility of unexpected disruptions to day-to-day life [1, 63], contrary to otherwise normative times [97].

E-commerce channels may provide a sense of normality [10, 16, 94] which can garner trust among consumers [63]. This may be particularly accentuated during the COVID-19 pandemic where consumers are more likely to be exposed to targeted advertising with increased exposure to online mediums of entertainment, news, and social media [84]. That is, when faced with the need to cope with uncertainty, consumers may be more inclined to trust online alternative channels of shopping to yield comparable hedonistic experiences as traditional modalities and subsequently utilize the service for their impulsive desires [40, 58]. Thus, we posit the following hypotheses:

#### Hypothesis 2

Perceived uncertainty of COVID-19 variants is positively associated with online shopping trust.

#### Hypothesis 3

Online shopping trust is positively associated with online impulse buying.

#### Hypothesis 4

Online shopping trust mediates the effect of perceived uncertainty of COVID-19 variants on online impulse buying.

### COVID-19 burnout & impulsive consumption

Burnout is defined as the psychological phenomenon of exhaustion, detachment, and feelings of inadequacy stemming from prolonged exposure to stressors [54]. Recently, studies have documented individuals experiencing *COVID-19 burnout* as a result of prolonged exposure to pandemic-related news, events, demands, and intrusive changes to daily life [98]. In contrast to the domain-general stress burnout, COVID-19 burnout is argued to be triggered from COVID-19-related thoughts and feelings [98]. COVID-19 burnout may have notable implications for online consumption, as e-commerce presents a readily available avenue for coping with pervasive pandemic-related stressors. Indeed, negative state is associated with the tendency to externalize symptoms through risky and impulsive behaviors, as observed in delayed discounting and gratification tasks [55, 93] as well as consumerism [4, 12, 87].

Because anxiety and worry represent reactance to negative stimuli, they counter self-control and result in both cognitive and affective exertion [65, 69]. The motivated regulatory failure perspective of *Ego-Depletion Theory* (EDT) [4, 5] suggests that prolonged exertion from negative states renders one less motivated to maintain self-control and increases the subjective reward value of hedonistic stimuli [34]. Under distress, consumers may be inclined to discard their self-control and long-term goals [53] in favor of impulsive and hedonic purchasing as a means of coping [4, 35, 65]. In other words, in the similar manner as domain-general stress burnout, it is likely that COVID-19-specific burnout will correspondingly result in low levels of self-regulation and high levels of impulsivity. For these reasons, we posit the following:

#### Hypothesis 5

COVID-19 burnout is positively associated with online impulse buying.

#### Hypothesis 6

COVID-19 burnout is negatively associated with self-regulation.

#### Hypothesis 7

COVID-19 burnout is positively associated with impulsivity.

### Self-control and the urge to buy

Self-control reflects one’s abilities to maintain regulatory focus and motivation against impulsive forces [4, 5, 34]. In practice, consumers with high self-control are less prone to impulse buying and exhibit more responsible management of fiscal spending within both real [4, 99, 73], and virtual spaces [89, 90]. Self-control and regulatory focus thereby facilitates the mitigation of reactance to adverse stimuli [13, 34]. Hence, online impulsive buying is a fundamental consequence of self-regulatory failure, yielding to temptations of trait impulsivity beyond other merited characteristics afforded by e-commerce (e.g., convenience) [35, 44, 73]. Thus, we identify self-control tendencies as potential moderators and posit the following:

#### Hypothesis 8

Self-regulation buffers (i.e., negatively interacts) the effects of a) perceived uncertainty of COVID-19 variants and b) COVID-19 burnout on online impulse buying.

#### Hypothesis 9

Impulsivity facilitates (i.e., positively interacts) the effects of a) perceived uncertainty of COVID-19 variants and b) COVID-19 burnout on online impulse buying.

Self-control, albeit often characterized as trait qualities, is malleable and directly responsive to external working forces. Building consumer self-control can shield against urges of online impulse buying [21, 81, 88], but bouts of impulsivity are nonetheless common occurrences for the majority of consumers [30, 74]. In historical accounts of disasters, such as in the aftermath of 2005 Hurricane Katrina or the 2011 Christchurch Earthquake, stressed consumers showed impulsivity in their buying habits and sought greater hedonic purchases [22, 77]. Further, consumers adaptively responded with increased consumption levels in anticipation of impending disasters that may strip one’s agentic control over their environment [4, 35]. For these reasons, we posit the following:

#### Hypothesis 10

Self-regulation is negatively associated with online impulse buying.

#### Hypothesis 11

Impulsivity is positively associated with online impulse buying.

#### Hypothesis 12

Self-regulation mediates the effect of COVID-19 burnout on online impulse buying.

#### Hypothesis 13

Impulsivity mediates the effect of COVID-19 burnout on online impulse buying.

## Current studies

**Fig. 1:**
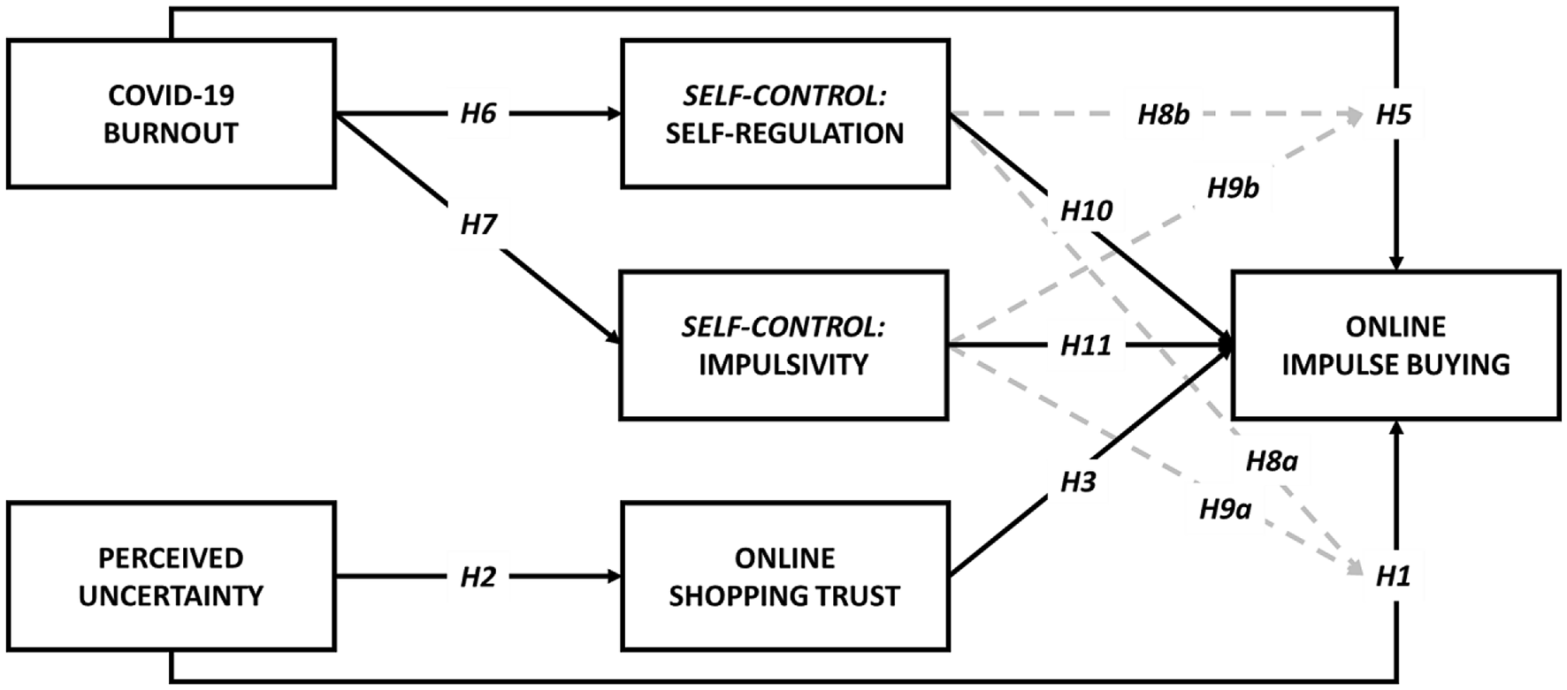
**Proposed conceptual mediated moderation model.** COVID-19 burnout and perceived uncertainty were added as covariates in the paths to all mediators. Additional covariates included respondent Age, Gender (Male, Female), Monthly Expenses, and Years of Experience in Online Shopping

The conceptual model with hypothesized paths is given in Fig. 1. We examined COVID-19 variants and online impulse buying in this study for two primary motives. First, as the COVID-19 pandemic had been ongoing for more than a year by the time of data collection, Chinese consumers may have developed familiarity with the original strain of COVID-19 thereby nullifying its associated novelty or uncertainty. However, in January 2021, a viral spread of variants in Hebei province marked the first prominent resurgence of COVID-19 in China since its initial outbreak in Wuhan. Secondly, data were collected as part of a larger study in two phases. The first phase of data collection occurred during the week immediately preceding the 2021 Chinese Lunar New Year—a major celebratory holiday period known for heavy consumerism among young adults—which was the return of festivities after the 2020 Spring Festival was cancelled amid rising cases of COVID-19. Although the week preceding the Spring Festival provided an opportunity to collect data during a relevant social ecology of high consumerism and COVID-19 variant fears, the effects observed may likewise be skewed for these reasons. Thus, the second phase of data collection occurred during the Spring Festival and the 2 following weeks to investigate the robustness of our findings following high consumer traffic.

We primarily sought to investigate a younger consumer base by directly targeting samples of university students in light of recent research documenting strong reactance to the developments of the COVID-19 pandemic among the young adults who may lack the psychological resources to adequately cope with sudden stressors [91, 95, 96]. Accordingly, university students have been shown to be at risk of resorting to impulsivity as a means of maladaptively coping with life stressors [48, 50, 51, 91, 92] and may likewise show similar patterns in response to COVID-19-related stressors as documented in past disasters [36, 77]. Further, a recent report found that more than 80% of university students in China in Tiers 1–4 cities had some form of part-time jobs as additional sources of income beyond allowances from family or customary gift money from older family members during the New Year holidays [79]. Coupled with the return of the country’s largest festivities, we rationalized that the rising trend of part-time employment and disposable income among young adult consumers alongside the increasing popularity of e-commerce in China offered an important and eager consumer demographic to investigate [14].

## Study 1

Study 1 took advantage of the unique window to examine online impulse buying during a time of novel variants as well as high demand for online consumerism in anticipation of the return of the Spring Festival and visiting families for gift exchanges. To examine the conceptual path model, we used variance-based partial least squares structural equation modeling (PLS-SEM) in R with the SEMinR v.2.3.1 [26, 72] defining the composite variables with reflective measurements (i.e., Mode A). In contrast to the more common covariance-based SEM (CB-SEM) technique, PLS-SEM maximizes the variance explained by differentially weighting indicators to its respective composite variables and is more suitable for predictive models of individual paths compared to a comprehensive theory confirmation model [26, 27].

### Method

#### Procedures & participants

To ensure safe research procedures amid rising COVID-19 cases, participants were recruited through electronic online mediums using convenience and partial snowball sampling methods: (1) the study was advertised and shared by academic staff and faculty members on popular social media platforms utilized by students (e.g., WeChat, QQ), (2) the study was shared to students by academic faculty members during their lectures, and (3) participants were offered the survey link to share among their classmates and friends. The study was anonymous and hosted electronically on SurveyStar (Changsha Ranxing Science and Technology, Shanghai, China). Participants provided informed consent and were free to withdraw from the investigation at any time. Participation was voluntary, and no compensation or incentives were given. The current study was approved by the first listed author’s institutional ethics committee.

A total of 1560 participants across China were recruited from February 4 to 9, 2021, in the first phase of data collection as part of a larger study. Twenty-two participants were removed from the final analysis for being ± 2 standard deviations (SDs) from the average survey completion time. A further 31 participants were dropped for completing the survey in less than 150 s. Lastly, 12 participants were dropped for being younger than 18 or not reporting age^1^ for a final sample of n = 1495 (n_undergraduates_ = 1408, n_postgraduates_ = 13, n_working professionals_ = 74). Participant demographic information is given in Table 1.

**Table 1:** Descriptive statistics for Studies 1 & 2

Variable	Study 1	Study 2
*Age*		
Mean	20.940	20.146
Standard deviation	3.345	3.443
*Gender*		
Male	24.6%	39.3%
Female	75.4%	60.7%
*Grade/Status*		
1st Year	24.9%	47.6%
2nd Year	32.0%	25.1%
3rd Year	24.3%	18.2%
4th Year	12.9%	9.0%
Postgraduate	0.9%	0.0%
Working	4.9%	0.1%
*Monthly living expenses*		
Less than 1000 Yuan	30.8%	17.0%
1001–1500 Yuan	54.0%	41.8%
1501–2500 Yuan	10.4%	26.9%
More than 2500 Yuan	4.7%	14.3%
*Experience in online shopping*		
Less than 1 year	6.0%	5.7%
1–3 years	37.6%	37.6%
3–6 years	41.1%	40.3%
More than 6 years	15.4%	16.4%

#### Measures

Due to several measures being altered to fit the social ecological context of the COVID-19 pandemic, confirmatory factor analyses (CFA) were conducted to examine structural validity of these measures. Goodness of fit statistics for the following measures are given in Table 2.

**Table 2:** Confirmatory factor analyses goodness of fit indices for studies 1 & 2

Model	*χ*^2^	df	CFI	RMSEA^†^	90% CI		SRMR	ECVI
					Lower	Upper		
**Study 1**								
Online impulse buying								
* Original 3-Factor*	285.451	6	0.925	0.177	0.159	0.194	0.060	0.211
* Six-item 1-Factor*	303.118	9	0.921	0.148	0.134	0.162	0.062	0.219
* Reduced 5-item 1 Factor*	107.166	5	0.969	0.117	0.098	0.137	0.032	0.085
Self-Control Model	135.086	13	0.969	0.079	0.067	0.092	0.051	0.110
Perceived Uncertainty	1.610	2	1.000	0.000	0.000	0.048	0.004	0.012
Online shopping trust*	–	–	1.000	–	–	–	–	0.008
COVID-19 Burnout	772.445	35	0.942	0.119	0.112	0.126	0.034	0.543
**Study 2**								
Online impulse buying	46.068	5	0.986	0.094	0.071	0.120	0.023	0.072
Self-control	216.274	13	0.935	0.130	0.115	0.146	0.092	0.267
Perceived Uncertainty	5.697	2	0.999	0.045	0.000	0.090	0.008	0.024
Online shopping trust*	–	–	1.000	–	–	–	–	0.013
COVID-19 Burnout	761.757	35	0.921	0.15	0.141	0.159	0.038	0.869

*df* = Degrees of freedom; *CFI* = Comparative fit index; *RMSEA* = Root mean square error of approximation; *CI* = Confidence interval; *SRMR* = Standardized root mean square residual; *AIC* = Akaike information criteria; *ECVI* = Expected cross-validation index. *Online shopping trust was measured using three indicators and thus, shows perfect goodness of fit. ^†^Note that RMSEA is positively inflated as model df becomes smaller [37, 38]. We therefore rationalize scale structural reliability by consulting other goodness of fit metrics and McDonald’s omega

##### Perceived uncertainty of COVID-19 variants (POC)

Perceived uncertainty of COVID-19 variants was measured via 4 items that were adjusted to fit the current pandemic [20]. Participants rated each item on a 7-point Likert scale (1 = *Strongly Disagree*, 7 = *Strongly Agree*).

##### COVID-19 burnout (CB)

COVID-19 burnout was measured via a 10-item scale [98]. Items were rated on a 5-point Likert scale (1 = *Never*, 5 = *Always*).

##### Online shopping trust (OST)

Trust for online shopping was measured via a 3-item scale [47]. Items were rated on a 5-point Likert scale (1 = *Strongly Disagree*, 5 = *Strongly Agree*).

##### Self-control scale (SC)

Self-control was measured with the 7-item Chinese version [50, 51] of measures of self-control tendencies [57]. The scale measured self-regulation and impulsivity. Each item was rated on a 5-point Likert scale (1 = *Strongly disagree*, 5 = *Strongly agree*). Due to low inter-factor covariance (Table 1; standardized estimate coefficient [*std est*] =  − 0.044, *p* = 0.142), self-regulation (SC-R) and impulsivity (SC-I) were treated as independent constructs in the following analyses.

##### Online impulse buying behavior scale (OIB)

To measure online impulsive buying tendency during the COVID-19 pandemic, we adapted and revised the 6-item Online Impulse Buying Behavior Scale (OIBBS; [70]. The scale contained three dimensions: 1) emotional buying, 2) uncontrollable urge, and 3) emotional response. Confirmatory factor analysis yielded poor fit following the original 3-factor structure in addition to being inputted as a 1-factor structure (Table 2). One item was dropped for poor loading (< 0.4), and the newly revised 5-item scale was fitted to a 1-factor structure (Table 2). All items were rated on a 7-point Likert scale ranging from (1 = *Strongly disagree*, 7 = *Strongly agree*).

### Results

PLS-SEM measurement model item loadings and English translated wordings are given in Table 3. All measures showed acceptable to good reliability through both McDonald’s ω and composite reliability *ρ*_C_. Average variation extracted (AVE) metrics also show good construct validity (i.e., > 0.5) [26], and no individual item showed sign of poor loading (i.e., < 0.4).

**Table 3:** Study 1 PLS-SEM measurement model

Path	STD LD	SE	*ω*	*ρ*_C_	AVE
**Perceived Uncertainty of COVID-19 Variants**			0.894	0.924	0.754
PUC1 The outbreak of COVID-19 variants is unpredictable	0.789	0.018			
PUC2 The potential impact of COVID-19 variants is uncertain	0.911	0.008			
PUC3 The extent of impact of COVID-19 variants are uncertain	0.913	0.008			
PUC4 The COVID-19 variants may break out again	0.853	0.013			
**COVID-19 Burnout**			0.947	0.954	0.675
*When you think about COVID-19 overall, how often do you feel…*					
CBO1 …tired?	0.764	0.014			
CBO2 …disappointed with people?	0.797	0.013			
CBO3 …hopeless?	0.832	0.013			
CBO4 …trapped?	0.862	0.012			
CBO5 …helpless?	0.897	0.008			
CBO6 …depressed?	0.901	0.007			
CBO7 …physically weak/sickly?	0.857	0.011			
CBO8 …worthless/like a failure?	0.828	0.011			
CBO9 …difficulties sleeping?	0.779	0.016			
CBO10 … “I’ve had it”?	0.670	0.021			
**Online Shopping Trust**			0.890	0.929	0.814
OST1 I think the image of shopping websites I frequently use is good	0.915	0.007			
OST2 I think the reputation of shopping websites I frequently use is good	0.927	0.006			
OST3 I trust the quality and service of products bought online	0.862	0.012			
**Online Impulse Buying**			0.855	0.894	0.629
*During the COVID-19 pandemic…*					
OIB1 …I would buy things online on an impulse	0.770	0.016			
OIB2 …I would often buy things spontaneously	0.657	0.025			
OIB4 …I would have a strong urge to buy things when shopping online	0.833	0.014			
OIB5 …I was happy and satisfied when I bought things online impulsively	0.871	0.009			
OIB6 …I enjoyed the fun of buying things casually	0.817	0.013			
**Self-Control (Regulation)**			0.667	0.909	0.769
SC1 I am good at resisting temptation	0.866	0.011			
SC2 People would say I have iron self-discipline	0.890	0.008			
SC3 I am able to work effectively toward long-term goals	0.874	0.010			
**Self-Control (Impulsivity)**			0.791	0.860	0.607
SC4 I do certain things that are bad for me, if they are fun	0.674	0.026			
SC5 Pleasure and fun sometimes keep me from getting work done	0.777	0.018			
SC6 I can't stop myself from doing something, even if I know it’s wrong	0.867	0.009			
SC7 I often act without thinking through all the alternatives	0.784	0.017			

English translations shown. Original measurement items were given in Chinese. See Table 5 for original Chinese wording

PLS-SEM structural path estimates are given in Table 4. All estimates toward the path ends were sufficiently powered (OST *R*^2^_adj_ = 0.084, SC-R *R*^2^_adj_ = 0.062, SC-I *R*^2^_adj_ = 0.048, OIB *R*^2^_adj_ = 0.233, 1 − *β* all 1.000) and showed no sign of multicollinearity (Variation Inflation Factor [VIF] from 1.008 to 1.194). Heterotrait–monotrait ratio of correlations (HTMT) also indicated no signs of problematic discriminant validity (HTMT all < 0.437). POC was positively associated with OST and OIB. CB was negatively associated with SC-R while positively associated with SC-I and OIB. OST, SC-R, and SC-I were all positively associated with OIB. SC-I and SC-R did not significantly interact with either POC or CB. There was a negative interaction between POC and SC-I. All three hypothesized indirect effects were significant; OST partially mediated the effect of POC on OIB, while both SC-I and SC-R partially mediated the effect of CB on OIB.

**Table 4:** Study 1 PLS-SEM Direct & Indirect Path Estimates

Path	*h*	*β*	*t*	95% CI		
				Lower	Upper	*p*
**Direct effects**						
POC → OST	*H2*	0.253	8.192	0.188	0.311	< .001
POC → OIB	*H1*	0.066	2.489	0.012	0.124	0.019
CB → SC-R	*H6*	−0.171	−5.547	−0.235	−0.108	< .001
CB → SC-I	*H7*	0.189	6.269	0.130	0.247	< .001
CB → OIB	*H5*	0.067	2.844	0.019	0.113	0.005
OST → OIB	*H3*	0.145	5.170	0.090	0.198	< .001
SC-R → OIB	*H10*	0.193	6.052	0.133	0.260	< .001
SC-I → OIB	*H11*	0.301	8.846	0.233	0.364	< .001
**Interaction effects**						
POC × SC-R → OIB	*H8a*	0.019	0.149	−0.063	0.120	0.719
POC × SC-I → OIB	*H9a*	−0.070	−1.625	−0.128	0.009	0.045
CB × SC-R → OIB	*H8b*	0.019	0.786	−0.110	0.124	0.779
CB × SC-I → OIB	*H9b*	0.046	0.920	−0.013	0.096	0.187
**Indirect effects**						
POC → OST → OIB	*H4*	0.037	4.263	0.021	0.053	< .001
CB → SC-R → OIB	*H12*	− 0.033	− 3.975	− 0.052	− 0.018	< .001
CB → SC-I → OIB	*H13*	0.057	5.317	0.037	0.078	< .001

*POC* = Perceived uncertainty of COVID-19 variants; *OST* = Online shopping trust; *CB* = COVID-19 burnout; *SC-R* = Self-control regulation; *SC-I* = Self-control impulsivity; *OIB* = Online impulse buying; *h* = Hypothesized path

### Discussion

Study 1 results generally supported our hypotheses but only showed one negative, albeit small, interaction effect; as impulsivity increased, the effect of perceived uncertainty of COVID-19 variants on online impulse buying became more negative. Although we hypothesized a positive interaction, where impulsivity will further promote the effect of uncertainty, one explanation for the contrary finding may be that impulsive individuals are motivated less by peripheral drivers and more by their own internal sense of impulsivity.

Further, those reporting high self-regulation tendencies surprisingly showed more online impulse buying in opposition to our hypothesized direction and implications from past research [4, 21, 68, 88]. One possible explanation is that individual reporting high self-regulatory tendencies has been exerting control over impulsive urges throughout the COVID-19 pandemic. In other words, these individuals may have already experienced self-regulatory collapse and motivational reprioritization from continuous exertion over the COVID-19 pandemic [34, 60], as illustrated from impulsive buying [4, 89], but may nonetheless report high self-regulation as would be otherwise properly reflective of one’s general perceived sense of self. However, because the current study did not measure specific behaviors, further research is needed to better capture possibility of regulatory failure.

## Study 2

Study 2 served to replicate and examine the robustness of Study 1’s findings with an independent set of respondents. As Study 1 took place immediately preceding the 2021 Spring Festival, Study 2 surveyed the young consumer base during the duration of the festival in addition to the weeks that followed to assess the consistency and robustness of our findings after high consumer traffic weeks.

### Methods

#### Procedures & participants

A total of 987 participants across China were recruited from February 12 to March 2, 2021. Eleven participants were removed from the final analysis for being ± 2 SDs from the average survey completion time. A further 27 participants were dropped for completing the survey in less than 150 s. Lastly, 16 participants were dropped for being younger than 18 or not reporting age^2^ for a final sample of n = 923 (n_undergraduates_ = 839, n_working professionals_ = 1). Participant demographic information is given in Table 1. The same procedures as Study 1 were followed for Study 2.

#### Measures

The same measures as Study 1 were used in Study 2. As was the case for Study 1, measurement structural goodness of fit metrics is given in Table 2.

### Results

PLS-SEM measurement model item loadings and original Chinese text wordings are given in Table 5. All measures showed acceptable to good reliability, good construct validity, and no individual item showed sign of poor loading.

**Table 5:** Study 2 PLS-SEM measurement model

Path	STD LD	SE	*ω*	*ρ*C	AVE
**Perceived Uncertainty of COVID-19 Variants**			0.930	0.948	0.822
PUC1 新冠肺炎及其变异病毒的爆发是无法准确预料的	0.839	0.018			
PUC2 新冠肺炎及其变异病毒造成的潜在影响是不确定的	0.949	0.007			
PUC3 新冠肺炎及其变异病毒的影响范围是不确定的	0.947	0.006			
PUC4 新冠肺炎及其变异病毒有可能再次爆发	0.884	0.017			
**COVID-19 Burnout**			0.955	0.961	0.713
*When you think about COVID-19 overall, how often do you feel…*					
CBO1 我感到疲劳	0.781	0.021			
CBO2 我对人们感到失望	0.832	0.020			
CBO3 我感到绝望	0.853	0.017			
CBO4 我感到陷入困境	0.892	0.012			
CBO5 我感到无助	0.925	0.008			
CBO6 我感到沮丧	0.916	0.009			
CBO7 我感到身体虚弱或生病	0.876	0.014			
CBO8 我感到自己一文不值或像个失败者	0.864	0.015			
CBO9 我感到睡眠困难	0.788	0.024			
CBO10 我会有 “我被感染了新冠肺炎”的想法	0.690	0.032			
**Online Shopping Trust**			0.916	0.944	0.849
OST1 我觉得常用购物网站形象好	0.947	0.005			
OST2 我觉得常用购物网站声誉好	0.948	0.006			
OST3 我信任网络购物产品的质量和服务	0.867	0.013			
**Online Impulse Buying**			0.896	0.921	0.701
OIB1 疫情期间, 我会在网络上购买计划之外的商品	0.807	0.019			
OIB2 疫情期间, 我买东西经常比较随意	0.680	0.031			
OIB4 疫情期间, 我网购时突然体验到要购买东西的强烈意愿	0.889	0.010			
OIB5 疫情期间, 在网络上购买计划之外的商品时, 我很高兴很满足	0.915	0.008			
OIB6 疫情期间, 我很享受随便购买东西的乐趣	0.874	0.011			
**Self-Control (Regulation)**			0.756	0.931	0.818
SC1 我能很好地抵制诱惑	0.916	0.008			
SC2 大家说我有钢铁般的自制力	0.891	0.011			
SC3 我能为了一个长远目标高效地工作	0.906	0.008			
**Self-Control (Impulsivity)**			0.816	0.873	0.634
SC4 我会做一些能给自己带来快乐但对自己有害的事	0.671	0.034			
SC5 有时我会被有乐趣的事情干扰而不能按时完成任务	0.799	0.022			
SC6 有时我会忍不住去做一些明明知道不对的事情	0.861	0.015			
SC7 我常常考虑不周就付诸行动	0.837	0.017			

Original Chinese measurement item wording shown. See Table 3 for English translations

PLS-SEM path estimates are given in Table 6. All estimates toward the path ends were sufficiently powered (OST *R*^2^_adj_ = 0.077, SC-R *R*^2^_adj_ = 0.030, SC-I *R*^2^_adj_ = 0.041, OIB *R*^2^_adj_ = 0.311, 1 − *β* from 0.991 to 1.000) and showed no sign of multicollinearity (VIF from 1.012 to 1.252). HTMT also indicated no signs of problematic discriminant validity (HTMT all < 0.423). POC was positively associated with OST and OIB. CB was negatively associated with SC-R and positively associated with SC-I but not OIB. OST, SC-R, and SC-I were all positively associated with OIB. SC-R did not significantly interact with either POC or CB. SC-I did not significantly interact with CB but negatively interacted with POC. All three hypothesized indirect effects were significant; OST partially mediated the effect of POC on OIB, while both SC-I and SC-R fully mediated the effect of CB on OIB.

**Table 6:** Study 2 PLS-SEM Direct & Indirect Path Estimates

Path	*h*	*β*	*t*	95% CI		
				Lower	Upper	*p*
**Direct effects**						
POC → OST	*H2*	0.243	5.674	0.157	0.326	< .001
POC → OIB	*H1*	0.088	2.639	0.024	0.155	0.009
CB → SC-R	*H6*	− 0.099	− 3.073	− 0.160	− 0.034	0.002
CB → SC-I	*H7*	0.198	6.568	0.139	0.256	< .001
CB → OIB	*H5*	0.029	1.023	− 0.028	0.084	0.328
OST → OIB	*H3*	0.162	4.749	0.092	0.230	< .001
SC-R → OIB	*H10*	0.289	7.215	0.213	0.371	< .001
SC-I → OIB	*H11*	0.299	7.668	0.220	0.375	< .001
**Interaction effects**						
POC × SC-R → OIB	*H8a*	− 0.009	− 0.113	− 0.084	0.057	0.795
POC × SC-I → OIB	*H9a*	− 0.108	− 3.086	− 0.171	− 0.033	0.002
CB × SC-R → OIB	*H8b*	− 0.045	− 0.699	− 0.128	0.135	0.488
CB × SC-I → OIB	*H9b*	− 0.017	− 0.444	− 0.151	0.148	0.857
**Indirect effects**						
POC → OST → OIB	*H4*	0.039	3.584	0.020	0.062	< .001
CB → SC-R → OIB	*H12*	− 0.028	− 2.861	− 0.048	− 0.010	0.004
CB → SC-I → OIB	*H13*	0.059	4.779	0.036	0.083	< .001

*POC* = Perceived uncertainty of COVID-19 variants; *OST* = Online shopping trust; *CB* = COVID-19 burnout; *SC-R* = Self-control regulation; *SC-I* = Self-Control impulsivity; *OIB* = Online impulse buying; *h* = Hypothesized path

### Discussion

Findings from Study 2 generally support findings from Study 1. However, COVID-19 burnout was not associated with online impulse buying. This finding suggests that burnout’s association with impulse buying may be more varied and responsive to environmental changes. Within the context of the current study, holiday festivities may have served to broadly reduce COVID-19-related burnout while still emphasizing consumerism amid celebration. Indeed, Welch’s t-test means comparison of COVID-19 burnout showed significantly higher burnout in Study 1 (pre-holiday; M ± SD = 1.821 ± 0.680) than Study 2 (holiday; M ± SD = 1.638 ± 0.671; M_dif_ = 0.183, *t*[1972.810] = 6.491, *p* < 0.001, *d* = 0.271). However, no difference was found for impulse buying between the two study samples (M_dif_ = 0.017, *t*[1816.071] = 0.355, *p* = 0.722, *d* = 0.015). The negative interaction between uncertainty and impulsivity remained robust with Study 1 but showed a notably stronger effect in Study 2.

## General discussion

### Uncertainty & trust’s association with online impulsive buying

The results of PLS-SEM from samples in phases 1 and 2 of data collection provide partial support of our hypothesized conceptual path model. Consistent with past implications [2, 25, 45, 82, 83], our findings add to the theoretical proposition that uncertainty drives individuals to minimize risk by opting for more consistent and predictable avenues both in isolated decisional tasks [17, 52] and in practice [33, 82, 83]. However, as the Chinese government utilizes a strict Zero-COVID policy [59], with sudden government-mandated lockdowns, the connection between uncertainty and online shopping trust will benefit from cross-cultural replication. For instance, for other countries where outlets for consumerism remain unchanged despite the pandemic, there may be no additional incentive for consumers to view e-commerce more favorably beyond convenience.

Perceived uncertainty of COVID-19 variants nonetheless remained a consistent positive, albeit weak, correlate of online impulse buying. This suggests that there are likely mechanisms activated by uncertainty not captured in the current model. Because uncertainty can trigger various negative affect valence [31, 82, 83], fear and anxiety are likely to also serve as mediating mechanisms in driving impulsive buying [12]. Given the novelty of COVID-19 variants at the time of the study, consumers may have assumed greater risks on the basis of the variants being plausibly, rather than likely, dangerous. Indeed, the current study’s analytical plan emphasized the predictive power of proposed conceptual antecedents but was not designed with the intention of confirming a broader theoretical model. The current study thus provides a foundation to investigate further the implications of uncertainty in risk mitigating and negative affect valence coping behaviors, such as online impulse buying.

### Burnout & self-control’s association with online impulse buying

COVID-19 burnout’s association with online impulse buying was statistically inconsistent across the two studies but nonetheless consistently weak in effect size magnitudes, suggesting that any apparent effect of COVID-19 on subsequent impulsive buying behaviors is likely to be substantively minor. Further, pre- and post-festival means comparison between Studies 1 and 2 showed a moderate drop in burnout. Celebrations may have minimized some degree of stress among participants that may have temporarily skewed the association between burnout and impulsive buying in Study 1. Nonetheless, we observed notably larger effects of COVID-19 burnout on both self-regulation and impulsivity, consistent with past studies in decision making [55, 93] and consumer behavior [4, 87]. These findings support the theoretical application of EDT [4, 5] and the revised motivation reprioritization perspective [34] on self-control during the COVID-19 pandemic. However, our findings did not support the original hypothesized direction [4, 35] that self-regulation is negatively associated with impulsive online shopping, instead showing the opposite.

As briefly mentioned before, one potential explanation for the contrary finding may be that consumers reporting high self-regulation were the types to have already been exerting relatively greater control than their impulsive counterparts, both continuously and periodically. In other words, should this be the case, these consumers ought to be similarly susceptible to ‘yield to their temptations’ as a byproduct of depleted resources and reprioritizing their motivation toward other higher priority goals [4, 34]. Indeed, the effect size of regulation on impulse buying in Study 2 (i.e., Phase 2 of data collection during and after the festivities) was notably larger than in Study 1. Such effects may partly reflect greater self-regulatory exertion amid festivities and familial celebrations that result in collapse of self-control.

Impulsivity was expectedly the largest and most consistent correlate of online impulse buying [19] and also interacted negatively with perception of uncertainty. As alluded before, this seemingly counterintuitive finding may be indicative that as general impulsivity trait increases, it dominates the effect of other distal drivers of impulse buying [35, 44, 73]. That is, impulsive consumerism is likely to simply reflect a natural behavioral manifestation of trait impulsivity at the highest level [4]. At low levels of trait impulsivity, however, consumers may require additional peripheral and conceptually distal incentives to drive impulsive consumption [35, 44, 73].

Both self-regulation and impulsivity mediated the effect of burnout on online impulse buying. These findings support our hypothesis that self-control is reactive to situational instances of negative environmental stimuli [4, 6]. Although some studies have regarded self-control characteristics as personality traits, our examined model with both mediating and moderating pathways suggest that regulatory tendencies and impulsivity may be suitably modeled as situationally reactive mechanisms beyond just stable individual characteristics during social ecologies with sudden and acute stressors. Thus, interventions that aim to mitigate the degree of psychological burnout and other negative mental health outcomes (e.g., anxiety, depression) commonly observed with the advent of novel public health crises may be beneficial for consumers’ online buying behavior.

### Theoretical and practical implications

Although several studies have examined consumerism amid COVID-19, the current study uniquely contributes to the body of literature by incorporating UAR and EDT in the same model. In doing so, we propose that the sudden changes to consumption amid the pandemic [11, 24, 61] among young adults may be partly attributable to the reactivity of self-control and consumer perceptions to COVID-19 related events, such as variant spread. Several surprising findings, however, have implications for future inquiry and theory development. Firstly, the positive association between self-regulation and online impulse buying implies the possibility that subjective appraisals of self-control may indirectly capture behavioral exertion in the absence of other measures that allow respondents to nuance between traits and behaviors. In the event that behavioral self-regulation only yields short-term benefits before collapsing into motivational reprioritization, but remains consistent in self-appraised regulatory abilities, it may be necessary to investigate whether both trait and specific behavioral items are necessary in future methodological approaches when examining EDT during prolonged exposures to stressors.

Secondly, the negative interaction between impulsivity and perceived uncertainty suggests a hierarchy of psychosocial drivers of consumption behaviors that may not always be mutually compatible. Thus, conceptually proximal drivers of impulsive consumption behaviors like self-control may override distal drivers, rendering them irrelevant should said proximal drivers be large in magnitude. Impulsive consumption may therefore be a behavioral symptom of self-control deterioration at intense levels rather than being a branching manifestation. Nonetheless, the current study’s limited examination of only perceived uncertainty and burnout as conceptually distal drivers of online impulse buying requires further research in incorporating additional conceptually relevant predictors.

From a practical perspective, market researchers and practitioners may benefit from capturing psychosocial perceptions of uncertainty and subjective experience of COVID-19 burnout in consumer surveys. Such readings may be reflective of broader and conceptually proximal issues of consumption impulsivity that could fluctuate with changing dynamics of public health crises. Thus, psychological and behavioral metrics may be incorporated with traditional attribute and performance measures (e.g., brand perceptions, product performance) to assess whether changes in quarterly product sales may be credited to temporary rises in consumer impulsivity rather than true supply demand. Online retailers are thus recommended to continuously monitor the development and progress of any public health crises and the reactivity of the consumer base.

### Limitations

Both conceptual and methodological limitations exist for the present study. Conceptually, although the current study proposes a sequential mediation model, causality cannot be established due to the cross-sectional design. Future research can opt to utilize experimental designs to further test the proposed downstream effects. Secondly, because the research was conducted in the context of Chinese culture, other cultures with different degrees of COVID-19 variant exposure may show varying results and require empirical verification.

Methodologically, all variables were assessed via self-report attitudinal measures, and not observed or recorded behaviors. Future research may seek to capture sudden spikes in individuals’ spending behaviors to make definitive claims related to impulsive consumption. Secondly, our recruitment procedures primarily targeted university students through convenience sampling with a small minority of working professionals recruited through snowball sampling procedures. Thus, the generalizability of the current study’s findings is limited and may not reflect older consumers who likely have greater disposable income and utilize less online channels than our current sample.

## Conclusions

The current study provides original insight into examining how consumers respond to the uncertainty of COVID-19 variants by adjusting their trust toward online shopping. Further, the findings also reveal how COVID-19 burnout can have consequential downstream effects on online impulsive buying via self-control failure. With the current research, we offer a preliminary, but much needed, investigation into how consumers adjust and adapt their buying behavior to the changing dynamic of the pandemic. Although e-commerce may continue to observe high demand compared to pre-COVID-19 times, marketers should remain wary and continue monitoring the consumer base to isolate true demand from impulsive buying in response to public health crises.

## Data Availability

The datasets used and/or analyzed during the current study are available from the corresponding author on reasonable request.

## Abbreviations

UAR: Uncertainty avoidance and reduction
EDT: Ego-Depletion theory
RMB: Renminbi (i.e., Chinese Yuan)
M: Mean
SD: Standard deviation
SE: Standard error
STD EST: Standardized estimate coefficient
STD LD: Standardized item loading
PLS-SEM: Partial least squares structural equation modeling
CB-SEM: Covariance-based structural equation modeling
CFA: Confirmatory factor analysis
VIF: Variance inflation factor
*ω*: McDonald’s omega
*ρ*_c_: Composite reliability (Rho C)
AVE: Average variance extracted
HTMT: Heterotrait–monotrait ratio of correlations
*χ*^2^: Chi-square
df: Degrees of freedom
CFI: Comparative fit index
RMSEA: Root mean square error of approximation
CI: Confidence interval
SRMR: Standardized root mean square residual
AIC: Akaike information criteria
ECVI: Expected cross-validation index
POC: Perceived uncertainty of COVID-19 variants
CB: COVID-19 burnout
OST: Online shopping trust
SC: Self-control
SC-R: Self-control (Regulation)
SC-I: Self-control (Impulsivity)
OIB: Online impulse buying

## Footnotes

1 Participants were dropped when they did not report age due to the inability to confirm and verify age of informed consent for research participation.

2 One participant was dropped due to reporting an ineligible age (i.e., > 200). This participant was dropped due to the inability to confirm and verify meeting the necessary majority age for informed consent in research participation.
